# Advancing Precision Medicine: A Review of Innovative In Silico Approaches for Drug Development, Clinical Pharmacology and Personalized Healthcare

**DOI:** 10.3390/pharmaceutics16030332

**Published:** 2024-02-27

**Authors:** Lara Marques, Bárbara Costa, Mariana Pereira, Abigail Silva, Joana Santos, Leonor Saldanha, Isabel Silva, Paulo Magalhães, Stephan Schmidt, Nuno Vale

**Affiliations:** 1 PerMed Research Group, Center for Health Technology and Services Research (CINTESIS), Rua Doutor Plácido da Costa, 4200-450 Porto, Portugal; lara.marques2010@hotmail.com (L.M.); b.c.211297@gmail.com (B.C.); mariana.m.pereira2097@gmail.com (M.P.); abigailsilva@outlook.pt (A.S.); jmdmsantos@hotmail.com (J.S.); leonorpessanha@gmail.com (L.S.); iumsmed@gmail.com (I.S.); 2 CINTESIS@RISE, Faculty of Medicine, University of Porto, Alameda Professor Hernâni Monteiro, 4200-319 Porto, Portugal; 3 Department of Community Medicine, Health Information and Decision (MEDCIDS), Faculty of Medicine, University of Porto, Rua Doutor Plácido da Costa, 4200-450 Porto, Portugal; 4 ICBAS—School of Medicine and Biomedical Sciences, University of Porto, Rua de Jorge Viterbo Ferreira 228, 4050-313 Porto, Portugal; 5 Department of Biomedicine, Faculty of Medicine, University of Porto, Rua Doutor Plácido da Costa, 4200-450 Porto, Portugal; 6 Coimbra Institute for Biomedical Imaging and Translational Research, Edifício do ICNAS, Polo 3 Azinhaga de Santa Comba, 3000-548 Coimbra, Portugal; paulo.r.magalhaes1@gmail.com; 7 Center for Pharmacometrics and Systems Pharmacology, Department of Pharmaceutics, College of Pharmacy, University of Florida, 6550 Sanger Road, Office 465, Orlando, FL 328227-7400, USA; sschmidt@cop.ufl.edu

**Keywords:** precision medicine, in silico, clinical pharmacology, computational tools, patient-centered healthcare

## Abstract

The landscape of medical treatments is undergoing a transformative shift. Precision medicine has ushered in a revolutionary era in healthcare by individualizing diagnostics and treatments according to each patient’s uniquely evolving health status. This groundbreaking method of tailoring disease prevention and treatment considers individual variations in genes, environments, and lifestyles. The goal of precision medicine is to target the “five rights”: the right patient, the right drug, the right time, the right dose, and the right route. In this pursuit, in silico techniques have emerged as an anchor, driving precision medicine forward and making this a realistic and promising avenue for personalized therapies. With the advancements in high-throughput DNA sequencing technologies, genomic data, including genetic variants and their interactions with each other and the environment, can be incorporated into clinical decision-making. Pharmacometrics, gathering pharmacokinetic (PK) and pharmacodynamic (PD) data, and mathematical models further contribute to drug optimization, drug behavior prediction, and drug–drug interaction identification. Digital health, wearables, and computational tools offer continuous monitoring and real-time data collection, enabling treatment adjustments. Furthermore, the incorporation of extensive datasets in computational tools, such as electronic health records (EHRs) and omics data, is also another pathway to acquire meaningful information in this field. Although they are fairly new, machine learning (ML) algorithms and artificial intelligence (AI) techniques are also resources researchers use to analyze big data and develop predictive models. This review explores the interplay of these multiple in silico approaches in advancing precision medicine and fostering individual healthcare. Despite intrinsic challenges, such as ethical considerations, data protection, and the need for more comprehensive research, this marks a new era of patient-centered healthcare. Innovative in silico techniques hold the potential to reshape the future of medicine for generations to come.

## 1. Introduction

The concept of tailoring medical treatments to a patient’s characteristics based on modern tools is relatively recent. About three decades ago, many scientists thought such an idea was utopian [1,2]. Historically, clinical decision-making relied on clinical experience and pathophysiology knowledge, following a “one size fits all” approach [1,3]. The Human Genome Project accelerated this paradigm shift, as the rapid development of affordable DNA sequencing methods facilitated targeted therapies, revolutionizing healthcare [2,3,4,5].

Modern medicine now integrates several technologies for precise identification and treatment The framework for successful clinical outcomes revolves around the “five rights”: administration of the right drug to the right patient at the right time, in the right dose, and through the right route of administration [6]. This approach, considering the patient’s medical history, genes, environment, and lifestyle, defines precision medicine. In 2011, the United States National Research Council’s *Toward Precision Medicine* defined precision medicine as the “tailoring of medical treatment to the individual characteristics of each patient (…) to classify individuals into subpopulations that differ in their susceptibility to a particular disease or their response to a specific treatment” [7]. This trending field is based on a healthcare model grounded on data, analytics, and information, yet it is often confused with personalized medicine due to their similar meanings. Personalized medicine, an older concept, considers the patient’s genetic makeup, beliefs, preferences, knowledge, and social context. However, the term “personalized” could be misinterpreted as implying the development of treatments uniquely tailored to each individual [8], leading to the preference for the term “precision medicine” by the US National Research Council. Many authors still question this definition, and it remains a subject of ongoing debate.

**Figure 1:**
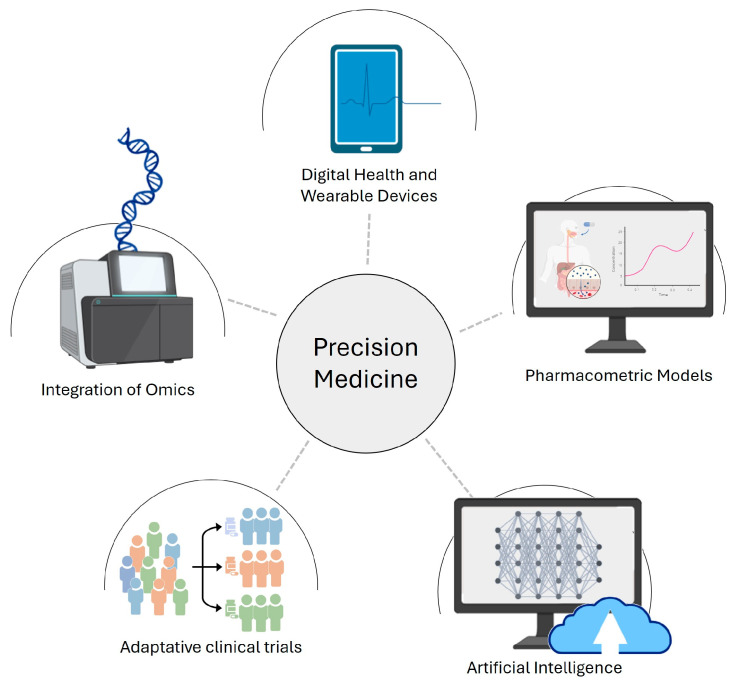
Key elements of in silico approaches in precision medicine. Created with Biorender.com. Available online: http://biorender.com/ (accessed on 12 October 2023).

Therefore, precision medicine addresses the growing need for precise and effective treatments, aligning with the cornerstones of the clinical medicine model, the four Ps: predictive, preventive, personalized, and participative [9]. This shift toward a patient-centered clinical decision-making system marks a transition from reactive medicine based on gold standards to patient-specific diagnostics and therapeutics [4]. In pursuit of robust precision medicine, in silico approaches have gained prominence, using computational methods to tailor therapies to individual patient characteristics (Figure 1). In this article, we aimed to present an updated review of in silico approaches, highlighting their impact on advancing precision medicine while spotlighting notable gaps and challenges within this field.

## 2. OMICS in Advancing Clinical Decision-Making

Advances in omics technologies since the discovery of the DNA structure have transformed precision medicine, offering unprecedented insights into the complex biological systems that underpin human health and disease. Omics, an umbrella term encompassing a set of biological fields such as genomics, proteomics, metabolomics, and other omics, which analyze the “omes” (the suffix comes from “chromosome”), refers to the collective technologies used to explore the roles, relationships, and actions of various molecules in an organism’s cells, significantly improving clinical decision-making, as they provide comprehensive insights into patient-specific molecular profiles, opening up new avenues for more precise prevention, diagnosis, and treatments [10,11,12]. While standard methods to study molecular mechanisms are time-consuming and proven to be inefficient, omics are structured on high-throughput analytical methods and have proven records of greater efficiency [13].

Drawing from our current understanding, precision medicine strives to deliver treatment to patients according to their own molecular characteristics (individual level) but also considers data related to the remaining population [14]. Its focus is on combining individualized data from patient-specific multi-omics with collective data in order to target the most suitable therapeutic strategy and founding structure for precision medicine in diverse populations [15]. Factors beyond genetic makeup, such as environmental influences and lifestyle, can also contribute to the complexity of predicting drug responses accurately.

The decision-making process related to precision medicine is usually driven by biomarkers as they may serve as indicators of a disease or a certain physiological state. In fact, research development of biomarkers is a current trend for pharmaceutical industries. As an example, the proteomics study of cancer is capable of revealing crucial information about the growth of the tumor and metastasis, leading to the identification of biomarkers and therapeutic targets [16]. The omics-based personalized medicine approach aims to discover biomarkers that provide highly detailed information about the pathology of the disease and therefore contribute to the decision-making process [17].

Considering the above, it means that clinicians should know how to interpret genomic data and biomarker results and apply them in current practice. Computer-based decision support (CBDDS) tools are able to offer support by listing the latest research and guidance and are required in order to aid clinicians through this process [18]. However, not all health care systems or clinicians are ready for this. A commitment to train personnel and to change the healthcare structure should be put in place to encourage precision medicine.

Indeed, there is a high hope within the scientific community regarding precision medicine and its power to improve the effectiveness of treatment and tolerability. However, before being applied in clinical practice, these studies typically undergo a complex process known as multi-omics. For example, the progression of a study involving multi-omics in metabolic diseases is usually followed by: (1) genomics for determining an individual’s complete genome and developing biomarkers; (2) pharmacogenomics to predict the treatment efficacy by analyzing genetic variants; (3) transcriptomics, studying external factors that influence gene expression and affect the patient’s phenotype; (4) epigenomics, to examine mechanisms that regulate gene expression; (5) proteomics, which focuses on studying protein function; (6) pharmacoproteomics, applying proteomics to pharmacology; (7) metabolomics, to identify metabolism variants; (8) pharmacometabolomics, applying metabolomics to pharmacology in order to support the development of personalized medicine by measuring metabolic phenotypes and drug metabolism; and finally, (9) integrating multi-omics, the integration and interpretation of the diverse omics, a complex exercise to apply in clinical routine [19].

### 2.1. Pharmacogenomics: Tailoring Treatment to Genetic Profiles

Pharmacogenomics stands at the forefront of precision medicine, integrating pharmacology and genomics to align medical treatments with the unique genetic makeup of each individual. The promise of pharmacogenomics lies in its potential to optimize drug therapy, minimize adverse drug reactions, and enhance treatment efficacy. By understanding the genetic variations that influence drug metabolism, transport, and mechanisms of action, healthcare providers can select medications and dosages that are best suited to an individual’s genetic profile [20]. Some examples include treatments for viral infections [21], oncology [22], and the choice of antidepressants [23] and heart disease medications [24]. For instance, pharmacogenomic testing for the HLA-B*5701 allele aids in identifying individuals at risk of hypersensitivity reactions to abacavir, improving treatment efficacy and safety [25]. In cancer patients, the testing for the thymidine synthase (TS) gene can help identify patients who may experience diarrhoea as a side effect of 5-fluorouracil (5-FU) chemotherapy. By adjusting the treatment plan based on the pharmacogenomic test results, healthcare providers can improve patient outcomes and reduce the risk of adverse effects [26].

Some common medications that require pharmacogenomic testing include: warfarin, because genes such as CYP2C9 and VKORC1 can help determine the most effective and safe dosage for an individual, reducing bleeding risks [27]; carbamazepine, because genetic testing for the HLA-B*1502 allele identifies those at higher risk of severe skin reactions, such as Stevens–Johnson syndrome [28]; and tamoxifen, because testing for the CYP2D6 gene can help identify individuals who may have a reduced ability to metabolize tamoxifen into its active form, allowing for personalized treatment plans to improve efficacy [29]. These examples illustrate how pharmacogenomic testing has been integrated into clinical decision-making to personalize medication choices and dosing, ultimately improving patient care and safety. As the field of pharmacogenomics continues to advance, it is expected that more medications and health conditions will benefit from personalized care guided by genetic testing.

Pharmacogenomics is an emerging and challenging field with limited clinical utility and applicability currently, but its impact is growing rapidly, with US Food and Drug Administration (FDA) approvals of personalized therapeutics involving biomarkers. Nonetheless, the clinical application of pharmacogenomics encounters substantial hurdles such as unknown validity across ethnic groups, underlying bias in healthcare, and real-world validation. Recent developments in the implementation of pharmacogenomics in personalized care include the Pharmacogenomic Clinical Decision Support System (PGx-CDS), which has been crucial in minimizing complexity and enabling clinicians to make informed medication decisions based on patients’ genetic profiles. There is a growing emphasis on the clinical implementation of pharmacogenomics, with proposed drug–gene pairs for implementation and the development of guidelines to integrate pharmacogenomic information into electronic health records (EHRs) and clinical decision support (CDS) systems.

### 2.2. Challenges and Considerations in Integrating OMICS: Navigating the Road to Precision Medicine

Progress in laboratory-based protocols, data storage, and bioinformatic capabilities has enabled the efficient generation of huge amounts of omics data in terms of both cost and time. This has been exemplified by the extensive COVID-19 research data generated within a few months [14]. However, omics methods, though widely employed in biomedical research, with several scientific studies being published in recent years, are still far from clinical reality. There are still many obstacles preventing translational-omics, a term that refers to the utilization of these new technologies in the clinical decision-making process [18]. Among these, maybe due to a lack of clinicians’ knowledge on this topic, there is a willingness of physicians to accept findings that primarily convey probabilities, such as the likelihood of disease presence or prognosis [14,30,31]. Furthermore, the large amounts of acquired data raise complex challenges, including the lack of technical knowledge to collect, handle, store, and transport samples, and limitations regarding multi-omics integration techniques. New creative approaches have been applied in this field. Machine learning (ML) and big data have been integrated with omics, leading to an improvement in the rapid and efficient collection, processing, and integration of vast amounts of data [30]. However, not all regions and healthcare settings have access to these advanced testing and interpretation tools.

Furthermore, the integration of pharmacogenomics and omics raises regulatory challenges, including standardization of testing methodologies and ensuring ethical use of genetic information. Navigating these regulatory landscapes is crucial for widespread adoption. Addressing these challenges requires ongoing research, technological advancements, and collaboration among researchers, clinicians, and policymakers.

The complexity of genomic medicine requires the development of guidelines and strategies for the integration of genomics into precision medicine. A structured clinical decision support system, combining clinical data and bioinformatics, is fundamental for this purpose [32]. Although CDS tools were created to guide clinicians to better integrate, use, and interpret genomic data, a recent study has shown that is still not clear what would be the best strategy [33]. Not only it is important to bring standard guidelines and strategies that can provide consistency in order to better train clinicians, but it is also important to promote an environment among different stakeholders (academics, clinicians, patients, government) to raise scientific awareness of the need and importance of creating more knowledge in precision medicine. Clinicians must be able to interpret this data for strong and effective decision-making; patients must be aware and well-informed to accept the treatment regimen they are prescribed; and the government must restructure the healthcare system, providing the necessary tools for this to become a reality in clinical practice. In fact, some of these measures are already implemented by regulatory agencies.

Until now, there has been a proven record that reflects the advances of omics in precision medicine which contributed to relevant discoveries. Several FDA-approved treatments now target individual characteristics of patients [34,35]. Among these, most are for the treatment of cancer (47%), rare diseases (37%), and other diseases (16%). The table below outlines some of the approved treatments for these therapeutic indications (Table 1).

According to the FDA, effective precision medicines require efficient tests able to aid diagnosis and a suitable treatment. These tests, or companion diagnostics, are named next generation sequencing (NGS) and are able to identify or sequence huge sections of patients’ genomes; therefore, they are considered a key advanced tool to be used in clinical practice [36]. The International Consortium for Personalized Medicine (ICPerMed) is another important international initiative to support precision medicine research [37]. Launched in 2016, it involves the European Commission and around 30 European and international members, including funders and policy-making organizations. ICPerMed, by conducting workshops and debates and providing reports on precision medicine implementation, aims to position Europe as a global leader in precision medicine research, actively promoting the science and demonstrating its societal benefits.

While omics technologies have significantly advanced the field of pharmacometrics, their contribution to precision medicine is not direct, but rather via the identification of relevant biomarkers. The high-throughput nature of omics technologies enables the fast discovery of candidate biomarkers, but their clinical validation and integration into precision medicine approaches require careful consideration of analytical development, computational modeling of the predictor, and clinical utility assessment.

## 3. Biomarkers and Molecular Diagnostics

**Figure 2:**
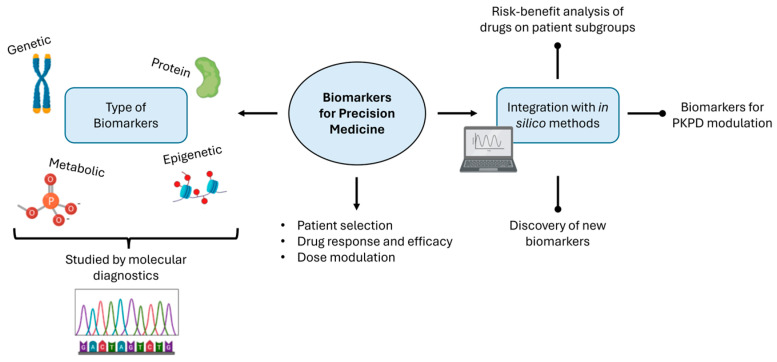
Biomarker integration in precision medicine and in silico approaches. Created with Biorender.com. Available online: http://biorender.com (accessed on 16 October 2023).

Having explored the transformative role of omics in advancing clinical decision-making, a pivotal component in this journey is the identification and utilization of biomarkers through molecular diagnostics. Biomarkers are biological observations, such as small molecules or clinical points, which are applied in drug discovery and used extensively in medical practice for screening, diagnosing, and characterizing diseases, as well as informing prognosis or therapy effects [38]. Biomarker analysis has started the shift toward an individualized treatment for each patient, and, as such, biomarker discovery is of high importance in this approach (Figure 2). New methods and techniques for efficient and quick analysis of biomarkers, surpassing the need for conventional monitoring, usually involve blood draws for imaging techniques [39].

The use of biomarkers in physiologically based pharmacokinetic (PBPK) modeling has shown promising potential in predicting drug responses and optimizing therapeutic strategies across diverse populations. These biomarkers offer valuable information about drug absorption, distribution, metabolism, and elimination (ADME) within an individual’s body, allowing for more precise drug exposure and response modeling. Additionally, biomarkers can help identify patient subgroups that are more likely to respond positively to drug therapy or those at higher risk of experiencing adverse events [40]. Incorporating pharmacokinetic (PK) biomarkers, such as drug concentrations, into research can enhance the accuracy and efficacy of drug development and personalized medicine.

Biomarkers are categorized based on their research and clinical practice roles. Genetic biomarkers, identifying genetic variations, play a crucial role in assessing disease risk and predicting treatment responses [41,42]. Protein biomarkers, such as enzymes, receptors, and cytokines, can indicate organ damage or dysfunction, abnormal cellular processes, or inflammation processes [43,44,45,46]. Metabolic biomarkers, derived from the analysis of metabolites and small molecules in various biological samples, provide valuable insights into metabolic pathways and can be used to identify markers of disease progression or treatment response [47]. Epigenetic markers, in turn, analyze alterations in DNA methylation, non-coding RNAs, or histone modifications, and samples can be obtained through either noninvasive or minimally invasive procedures, making these markers accessible, stable, and frequently chosen. They have been found to be very useful for cancer, as there are known epigenetic changes in the shift from somatic to cancerous cells [48]. One such example is the panel of promoter hypermethylation of the RASSF1A, RARβ2, and APC genes in serum and urine, which has a 94% specificity and sensitivity in detecting renal cell carcinoma [49].

The analysis of these biomarkers can be performed through molecular diagnostics, which encompasses several technologies that allow the study of genomic and proteomic biomarkers [50]. Essential assays include sequencing-based and non-sequencing methods, like immunohistochemistry (IHC), microsatellite instability testing (MSI), and chromosomal microarray analysis (CMA). Beyond those important tools for molecular diagnostics and biomarker analysis, they also include polymerase chain reaction (PCR), Sanger and NGS, and emerging techniques like liquid biopsies, RNA sequencing, and long-read sequencing [51].

Human PK investigations and clinical trials are the mainstays of conventional drug development procedures. However, these traditional methods frequently fall short of accurately portraying the wide variety of patient groups found in real-world situations. This restriction is particularly important since drug reactions might differ significantly between people due to their specific demographic, genetic, and environmental characteristics [52]. Generalized pharmacometric modeling (GPM) stands out as an effective remedy to these problems by integrating ML approaches with sizable, diverse datasets into the renowned framework of pharmacometrics methods, improving our understanding of drug disposition and its effects across a wide range of distinct patient groups. GPM might revolutionize the individualization of drug therapy by finding instructive and pertinent patient-specific variables, with a strong focus on biomarkers that affect drug dynamics [53]. Treatment plans that are customized for certain patient populations may result in safer and more efficient drug regimens [54]. GPM makes use of the capabilities of ML algorithms, particularly random forest regression [55] and Bayesian networks [56], to handle and comprehend vast and complex datasets, which traditional models sometimes find difficult to process, and unveil intricate connections and interactions between variables that could not have been seen otherwise. The biological and therapeutic significance of the GPM-identified biomarkers is an important topic for debate. Surprisingly, GPM occasionally reveals unanticipated biomarkers that appear to have no connection to drug dynamics but that are strongly grounded in the current scientific literature and can actually affect certain dynamics. The identification of biomarkers influencing the inter-individual variability in drug pharmacokinetics/pharmacodynamics (PK/PD) may be made easier with the use of this new knowledge, which will eventually lead to the development of more individualized and successful pharmacological regimens [53].

### 3.1. Harnessing Biomarkers for Precision Drug Development and Treatment Optimization

Biomarkers can be used for the development of in silico PKPD models of enzymatic activity. For example, a study aimed to assess the induction or inhibition of the cytochrome P450 (CYP450) enzyme CYP3A4 by using the biomarker 4β-hydroxycholesterol (4βHC) [57], which is directly associated with CYP3A4 activity [58]. The researchers applied a Bayesian technique for parameter estimation to develop the PKPD model [59,60], which predicts a differential impact of rifampin and ketoconazole on 4βHC and midazolam (MDZ), the industry standard CYP3A4 inhibitor detectors. Despite limitations, the PKPD model holds promise for precision medicine, allowing tailored prescription regimens, predicting drug–drug interactions (DDIs), and reducing negative consequences through early identification of CYP3A4-related dynamics [57].

The study of biomarkers using ML can allow benefit–risk analysis of drugs in various patient subgroups, enabling predictions of efficacy and adverse effects [61]. A study exemplified this approach by utilizing the random forest algorithm to analyze clinical trial results of acute melanoma patients treated with nivolumab to establish a relation between nivolumab clearance and several cytokines [62]. The researchers were able to establish a panel of biomarkers using the 16 top inflammatory cytokines that, even without the use of the drug, could be related with clinical benefit. Moreover, these biomarkers were able to predict nivolumab clearance, which is related to overall survival (OS) [63]. Effectively, this algorithm could predict OS by the clearance, in which patients with high clearance have decreased OS. This allows the stratification of patients in high and low clearance groups, enabling the prediction of treatment outcomes and an informed choice of treatment [64].

New biomarkers can also be discovered using in silico techniques. Radiomics collects digital medical images from magnetic resonance (MR), computed tomography (CT), positron emission tomography (PET), and other imaging techniques and transforms them into mineable data that can be quantitatively linked with pathophysiology. This effectively creates new imaging biomarkers that, when combined with patient characteristics and even genomic data, can help inform diagnostics, prognosis, and response to therapy, the basis of precision medicine [64]. Radiomics information can fundamentally reshape the development of pharmacometrics models, particularly in understanding the dynamics of tumor size. These cutting-edge pharmacometrics models shed light on the complicated interplay within a tumor ecosystem by accounting for the numerous characteristics of tumor heterogeneity both within and between lesions, and an example of such a model is the classification clustering of individual lesions (CICIL) methodology [65]. Still in the oncologic area, by drawing conclusions from radiomic data, this method can also be useful in understanding resistance mechanisms and identifying new biomarkers for drug resistance [66]. In a recent study, pharmacometric models were employed to explore the potential of biomarkers in predicting the efficacy of brazikumab, an anti-interleukin 23 monoclonal antibody [67], in the treatment of Crohn’s disease (CD). Two predictive biomarkers emerged from this study, baseline IL-22 (BIL22) and baseline C-reactive protein (BCRP), whose higher baseline levels represent a notably enhanced response to the drug [68]. Moreover, the study unveiled a strong negative correlation between the placebo effect and the baseline Crohn’s Disease Activity Index (BCDAI) [69], serving as a prognostic biomarker. Recognizing that the impact of the placebo effect is vital for interpreting clinical trial results accurately and refining treatment strategies accordingly, a correlation between high BCDAI and low clinical response to drugs has been found previously in the literature [70]. The pharmacometrics analysis also established quantitative cutoff values for BIL22 and BCRP, offering precise thresholds for patient stratification, a significant departure from traditional median-based cutoffs. Such precision in patient selection can pave the way for more effective clinical trial designs and enhance the likelihood of success in future studies. Furthermore, the study highlighted the superiority of pharmacometrics modeling over conventional statistical analysis. Its capacity to integrate longitudinal data while considering various sources of variability, including drug PK and placebo effects, proved to be a powerful tool for elucidating biomarker-dependent responses in biologic therapies [68].

### 3.2. Challenges in Implementing Biomarkers and Molecular Diagnostics in Precision Medicine

Thus, biomarkers and molecular diagnostics offer significant promise in healthcare, enabling early disease detection and personalized treatment. However, their effective implementation faces challenges that warrant attention. These include ensuring the accuracy and reliability of biomarker tests, which often require complex validation processes; standardization, as variations in measurement and interpretation can lead to inconsistent results across different laboratories; time related to regulatory approval; and ethical concerns surrounding informed consent, data privacy, and genetic discrimination, which require careful consideration. Clear guidelines for interpretation, addressing limited biomarkers, and integrating data effectively are crucial.

There have been some collaborations between academia, industry, and regulatory bodies to accelerate biomarker discovery and establish standardized approaches for biomarker validation for precision medicine. The European Medicines Agency (EMA) emphasizes early engagement with biomarker developers through various platforms like the Innovation Task Force, the Qualification of Novel Methodologies procedure, and the Scientific Advice procedure [71]. The EMA’s Regulatory Science Strategy includes measures to facilitate regulatory qualification for biomarkers. The FDA has a Biomarker Qualification Program (BQP), which is a voluntary process that allows biomarker developers to submit their data and information for FDA review and qualification as drug development or regulatory tools. The FDA also collaborates with other stakeholders through consortia, such as the Biomarkers Consortium and the Critical Path Institute [72].

The Innovative Medicines Initiative (IMI), a public–private partnership between the European Union and the European Federation of Pharmaceutical Industries and Associations (EFPIA), supports several projects such as PRECISESADS, AETIONOMY, RHAPSODY, and CANCER-ID [73], focusing on biomarker discovery, molecular mechanisms of non-response to treatments, and the development of tools, standards, and approaches to address unmet medical needs for effective disease-modifying treatments.

## 4. Pharmacometrics Tools: Significance and Challenges in Precision Medicine

Pharmacometrics is a critical tool in the field of clinical pharmacology, providing a quantitative framework for understanding, characterizing, and predicting drug exposure and response. The integration of omics and biomarkers is fundamental to pharmacometrics, providing vital quantitative data for characterizing and predicting drug behavior, ultimately enabling the optimization of therapeutic strategies and the development of precision medicine approaches. Therefore, drug development and optimization have become increasingly challenging over the years due to the diversity of compounds and therapeutic targets, the inherent variability in patient responses, and the evolving landscape of regulatory agencies in the drug approval process. Pharmacometrics has emerged as a multidisciplinary scientific discipline to address all these challenges, employing advanced mathematical and statistical methods based on biology, pharmacology, and physiology to quantify the interaction between the drug and the patient [74,75,76,77,78].

The introduction of the concept of pharmacometrics dates back to the 1960s, with the quantification of PK data in laboratory experiments and the development of methods to connect to pharmacodynamics (PD) [79,80,81]. Sheiner and Stuart Beal are the pioneers in this field, having created the Nonlinear Mixed Effects Modeling (NONMEM) software system in the 1970s, particularly well-known for its applications in population pharmacokinetic (popPK) studies [82], allowing the characterization of individual PK profiles and sources of variability in a population. From 1980 onwards, drug regulatory authorities, namely, the FDA, began to endorse the practice of pharmacometrics and, since then, this area has had a high impact on decisions related to clinical trial design, drug development, approval, and therapeutic regimen optimization [83]. Indeed, the pharmacometrics resources enable tailoring the therapeutic plan—the most appropriate drug dose and dosing schedule—for an individual patient based on factors such as genetics, age, weight, and underlying health conditions. In the preclinical phase, modeling and simulating the drug’s behavior in different patient populations allow for the design of clinical trials more efficiently, reducing costs and the time required in the drug development process [83]. Pharmacometrics tools also empower the prediction of different patient responses to a drug based on their unique characteristics, thus enabling the identification of biomarkers and patient subgroups that may benefit more or less from a specific drug [84,85,86]. Consequently, this allows for quicker decision-making regarding safety and efficacy, streamlining the evaluation and approval process of new drugs. Additionally, therapeutic regimen optimization also relies on modeling. This field has been supported by increasingly complex mathematical models, assuming a pivotal role in the era of precision medicine [87,88].

Initially met with skepticism, pharmacometrics faced challenges in integrating diverse data types for precision medicine research, slow adoption due to limited understanding, regulatory hurdles aligning with FDA requirements, and computational challenges in developing advanced models. Overcoming these required collaboration among clinicians, researchers, bioinformatics specialists, and biostatisticians. As the field has evolved, researchers, regulatory authorities, and funding bodies have recognized the power of pharmacometric analyses in improving pharmacotherapeutic use, drug development, and regulatory decisions. As the field continues to advance, it is essential to address these challenges and solidify pharmacometrics’ role in the era of precision medicine.

### 4.1. A Triad of Precision: PKPD, PBPK, and Population PK Models in Pharmacological Insights

PK and PD modelling, PBPK modelling, and popPK modelling are all tools used in pharmacometrics to understand drug behavior and optimize therapeutic strategies. PKPD modelling interrelates PK (ADME) and PD (effect on patients). PD models focus on concentration–effect relationships and are often integrated with PK modelling to optimize drug efficacy and minimize adverse effects [89,90].

The primary basis for PD models is concentration–effect relationships [91]. To our knowledge, there is a limited body of research exclusively focused on PD modelling, although some approaches may be outlined: simple direct effect models, biophase distribution, indirect response models, signal transduction models, and irreversible effect models [92,93]. Typically, these techniques are integrated with PK modelling, allowing for the characterization of the dose–exposure–response relationship, which is a crucial step in optimizing the drug efficacy and minimizing adverse effects, ultimately leading to improved therapeutic outcomes. Lin et al. [94] developed a population-based PKPD model for carfilzomib in adult patients with relapsed/refractory diffuse large B-cell lymphoma using the NONMEM^®^ software (version 7.4.1). Such studies contribute to ongoing research aiming to identify characteristics of patients who benefit from this specific treatment. Additionally, the relevance of PKPD model integration in drug development has also been stated. For instance, Palmer et al. [95] reviewed the implementation of PK and PD studies in antimicrobial drug development. Derendorf et al. [96] have also demonstrated that corticosteroids represent a class of drugs suitable for PKPD modelling studies, allowing the prediction of the systemic activity of novel corticosteroids based on their PK profiles. According to Zou et al. [89], this model technique also finds extensive application in drug delivery systems and the modification of large molecules, both in preclinical and clinical trials, providing essential insights for animal-to-human translation and facilitating the selection of therapeutic regimens. Even at the initial stages, during the discovery of novel compounds phase, these strategies can be effectively implemented [97]. PKPD model-based analysis enables a faster in vitro to in vivo translation, reduces the number of animal studies, and improves bench-to-bed translation. As evident, PKPD models offer a broad spectrum of applications, spanning from preclinical drug assessment to drug optimization, maximizing the patient’s therapeutic response.

PK itself represents a powerful tool to characterize the kinetic profile of several drugs. To explore the effects of the human body on a drug, specifically, to analyse its PK data, there are two common approaches: compartmental PK analysis and noncompartmental PK analysis (NCA) [98,99,100,101]. In the first method, the human body is conceptualized as a finite number of interconnected and kinetically homogenous compartments (representing various parts of the body, such as blood, organs, and other tissues), assuming that the rate of transfer between compartments and the rate of drug elimination from compartments follow first-order or linear kinetics. In turn, NCA is a simpler method that does not rely on specific compartmental models. Instead, it estimates PK parameters directly from the observed concentration–time data through algebraic equations. These analytical approaches have proved specific utility in advancing the development of complex drug delivery systems, namely, nanoparticles. Recently, Osipova et al. [102] compared two nanoparticle formulations using both NCA and compartmental analysis. Their findings underscored the potential of compartmental analysis to provide valuable insights into a crucial step in drug development, such as drug delivery.

Among the various PK modeling approaches, the most common mathematical models are popPK models and PBPK models. They are complementary techniques that scientists often use and are instrumental in the era of precision medicine [81]. There are a variety of software packages available to analyze, model, and simulate pharmacological data, including NONMEM^®^, Phoenix^®^ WinNonlin^®^, Simcyp, MATLAB^®^, GastroPlus, and Monolix. Most of these programs are user-friendly for scientists from diverse disciplines, but they are mainly used by experts in the field [103]. Based on evidence that concentrations of chemical substances within target tissues hold greater predictability for biological responses than external doses, the pre-eminence of PBPK modelling has increased significantly [104,105]. The concept of employing multicompartmental models that incorporate biological and physiological components to simulate PK data was originally introduced by Teorell in the 1930s [106]. Over subsequent decades, the number of publications involving PBPK models has increased significantly, demonstrating the growing interest in the implementation of this approach in the pharmaceutical industry, from the drug discovery and development process to post-market drug optimization [107,108].

PBPK models, which mechanistically describe drug disposition within the body by simulating ADME processes, have a huge focus on DDI. In a study conducted by Umehara et al. [109], the efficacy of a robust PBPK model in accurately predicting DDI was demonstrated. It can help reduce the number of DDI clinical trials. Previously, we have also highlighted the valuable utility of this tool within this field [110]. Specifically, we have developed a PBPK model of salbutamol and fluvoxamine to simulate the interaction between both drugs in different regimens and under diverse patient profiles.

Furthermore, the prediction of drug behavior in different populations and under varying physiological conditions, namely, age, ethnicity, or disease status, is easily conducted with PBPK models. Zamir et al. [111] assessed the PK of metoprolol in distinct cohorts comprising healthy, chronic kidney disease (CKD), and acute myocardial infarction (AMI) patients through PBPK modelling. Their findings led to the recommendation of metoprolol dosage adjustments at various CKD stages, along with the elucidations of PK differences in this β-blocker between the different subgroups. PBPK modelling can also predict drug disposition during pregnancy. For instance, Amaeze et al. [112] developed a PBPK model to evaluate N-acetyltransferase 2 phenotype-specific effects of pregnancy on isoniazid disposition. Hence, tailoring dosage strategies for vulnerable groups is increasingly becoming a reality.

PopPK modelling involves examining PK on a broader scale, where data from all individuals in a population are simultaneously analyzed using a NONMEM. The development of a popPK model encompasses five key elements: the data, structural model, statistical model, covariate model, and modeling software. Mould et al. detail all these aspects [113]. Similar to all the aforementioned approaches, popPK models have wide applicability in the pharmaceutical industry, and an increasing number of studies are being conducted. In particular, the optimization of therapeutic regimens along with the identification of patient characteristics (also referred to as covariates) with an impact on drug kinetics has been the core of this area. There has been a growing use of popPK models to study various diseases across diverse areas. Researchers are now employing retrospective studies using real-world data to examine relationships between covariates and drug PK parameters [114]. Other studies are being developed within clinical trials. For instance, a study used PK data from an open-label, randomized, two-treatment, two-period, two-sequence, single oral dose, crossover, bioequivalence study to develop a popPK model for simulating various doses of a recently produced drug for acute lymphoblastic leukemia (ALL), aiding in selecting the bioequivalence dose. Some authors also focus on addressing significant gaps in clinical knowledge. For example, the impact of renal function on amisulpride PK was examined using popPK modeling by Li et al. [114]. A popPK model of dasatinib developed by He et al. [115] evidenced that low doses should be recommended for Chinese patients.

Currently, researchers have at their disposal a modelling continuum, ranging from popPK to quantitative systems pharmacology (QSP) models. QSP models integrate complex biological pathways and drug interactions to predict responses to drug interventions. For example, a QSP model might be used to understand the impact of a new cancer drug on various signalling pathways involved in tumor growth. A recent study aimed to assess if targeting regulatory T cells (Treg) could enhance the efficacy of a checkpoint inhibitor (anti-PD1) in inhibiting tumor growth. Mice experiments alone were insufficient in providing insights into longitudinal changes in biomarkers. QSP modelling was employed to elucidate the mechanistic interplay of anti-PD1 and anti-Treg on Treg and effector T cell (Teff) longitudinal changes. The QSP model focused on essential components characterizing major pathways in the immune system. It represented the dynamics through Teff (cytotoxic effector T cells), Treg, and PD1/PDL1, linking these components to tumor growth modulation. The findings suggest that Teff profiles may be more predictive of pharmacological responses than Treg profiles, which provides valuable insights for drug development decisions in immuno-oncology [116].

The decision of which modeling approach to use should be based on the specific goals of the research or application, considering factors like data availability and system complexity.

### 4.2. Challenges of Quantitative Drug Modeling

Pharmacometrics mainly relies on data, and the quality and quantity of available data can vary significantly. Poor quality data collection compromises model development and validation, leading to less accurate predictions. For example, popPK models may not be well supported by commonly used sparse sampling because of the slow absorption and long half-life of some drugs [117]. In fact, scientists with training and expertise in quantitative drug modelling may face some concerns when it comes to collecting PK data. While PK data is typically gathered during early clinical trials, the number of data points collected per subject is quite limited in phase II and III studies due to ethical and medical considerations, resulting in sparse PK sampling [118]. This constraint also applies to pediatric studies, where efforts are made to minimize the volume of blood sampled. In addition, data collection often lacks accurate time information, leading to measurement errors (ME). Choi et al. [119] have highlighted that time ME can lead to bias in parameter estimators since the time variable used in PK modelling differs from the actual collection time. To address this, the authors have proposed two methods for correcting time ME: in cases where the PK profile exhibits minor curvature, conventional population PK modeling can be employed; however, in scenarios where the curvature is moderate or large, the most reliable approach is the transform both sides (TBS) model, which preserves a nonlinear relationship between response and structural variables such as time, ensuring that PK parameters maintain their original interpretation. Another innovative approach involves the use of dried blood samples through wearable automatic sampling systems rather than conventional blood sampling techniques. In a study conducted using blood samples from Beagle dogs [120], these novel systems demonstrated the capability to yield a higher number of samples, allowing the collection of more PK data, representing a promising alternative to established methods.

Traditionally, parameter estimation in PK modelling relied heavily on mathematical equations and assumptions about physiological processes. However, these classical methods often struggled to capture the complexities and inter-individual variations in drug PK. Data-driven approaches offer a paradigm shift by leveraging vast datasets, advanced computational techniques, and ML algorithms to improve parameter estimation. Traditional parameter estimation methods in PK can include the standard two-stage (STS) approach, which involves fitting a PK model to individual data, estimating individual PK separately for each individual, and then combining the individual parameter estimates [121]. The naive pooled data (NPD) approach involves fitting all individuals’ data together as though there were no individual kinetic differences [122]. Furthermore, the Bayesian estimation method provides a powerful approach to individualizing dosing regimens. It incorporates elements of variability in previously known population estimates and variability in the PK parameters and known errors intrinsic to the assay method used to estimate the blood fluid drug concentrations [123]. However, these traditional methods have some limitations. They often require distributional assumptions and model linearization. They may encounter issues with local minima and underdetermined problems [124]. They are based solely on the plasma concentrations obtained from individual patients and applied directly to PK equations [123].

In addition, model evaluation represents a significant hurdle in pharmacometrics. Generally, models can be evaluated internally or externally [125]. Internal evaluation involves basic methods such as analyzing goodness-of-fit (GOF) graphs to detect potential biases or problems in the structural model, as well as the evaluation of the accuracy of parameter estimates from standard errors or confidence intervals. Advanced methods include data splitting, resampling techniques, or Monte Carlo simulations (visual predictive check). External evaluation, which is not very common, involves comparing a validated dataset with the predictions obtained from the built model. For this reason, ensuring an accurate predictive model is challenging.

Nonetheless, we also face ethical issues since dose-finding PK studies may not provide direct participant benefit, posing the dilemma of balancing individual research-associated burdens with intended long-term benefits [126,127]. There are also difficulties in recruiting peer-reviewers with appropriate modelling expertise and experience, lack of confidence in PBPK models for which no tissue/plasma concentration data exist for model evaluation, lack of transferability across modelling platforms, poor in vitro–in vivo correlations, and knowledge gaps in system parameters [128,129].

## 5. Data Integration and Analytics: Data-Driven Approaches in Pharmacokinetic Modeling

The demand for precision dosing of established medications post-approval has become routine in the evolving healthcare landscape. As aforementioned, mathematical modelling is a valuable tool that extends its utility beyond late-stage clinical development [130,131]. Healthcare professionals, including clinicians and providers, increasingly recognize the significance of patient-specific responses to standardized dosing protocols [132,133]. This awareness holds particular importance in the context of diseases such as cancer [134], human immunodeficiency virus (HIV) [135], and tuberculosis (TB) [136], where drug PK variability can profoundly influence treatment outcomes.

### 5.1. Unraveling Complexity: Data-Driven Pharmacokinetic Modeling in Combination Therapy

**Figure 3:**
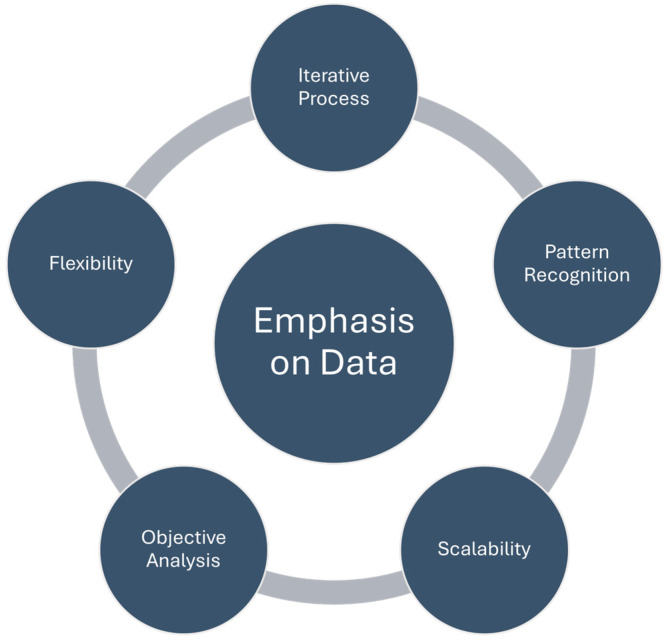
Key characteristics of data-driven approaches.

In the context of data integration and analytics, these diseases benefit from the ability to gather and analyze vast amounts of data. When modelling monotherapy, the focus is primarily on understanding the PK and PD of a single drug to predict its behavior and efficacy. In contrast, modelling combination therapy involves the complex interplay of multiple drugs, each with its own PK properties, mechanisms of action, and potential for DDIs. Data integration and analytics became crucial for assessing how these drugs work together. In data-driven PL modelling for combination therapy, researchers and healthcare professionals need to consider the additional layers of complexity introduced by multiple drugs. This complexity underscores the importance of data integration and analytics in tailoring treatment regimens to individual patients effectively. The goal is to achieve the best possible therapeutic outcomes while addressing the unique challenges posed by these complex interactions (Figure 3).

Access to a growing volume of patient-specific digital health data and an expanding pharmacological and disease-specific pathophysiological knowledge presents new opportunities. This data, often beyond the scope of traditional clinical trials, can be used to create models that support clinical decision-making, leading to enhanced patient care and improved treatment outcomes [137]. As previously stated, precision medicine relies on integrating and analyzing diverse datasets to understand individual patient profiles, identify biomarkers, and tailor treatments. This step involves merging datasets from various sources, including EHRs, genomic data, proteomic data, and other “omics” data (e.g., metabolomics), to derive meaningful insights that guide personalized healthcare decisions. The integration of omics and EHR data is particularly important, as it allows for a more comprehensive understanding of a patient’s health status and treatment history. For example, EHR data can provide information on a patient’s medical history, medication use, and clinical outcomes, while omics data can provide information on genetic variations, protein expression, and metabolic profiles. Nevertheless, standardized data formats and interoperability between different data sources (as EHR data is often stored in different formats and systems) and privacy concerns and regulatory requirements represent some of the challenges encountered. Despite this, the integration of omics and EHR data holds great promise for advancing precision medicine [138,139].

Advanced analytics methodologies, including ML and artificial intelligence (AI), are pivotal in making sense of integrated datasets [140]. These technologies can identify patterns, correlations, and predictive insights that might be challenging to uncover through traditional methods. Predictive models can be developed to forecast disease risk, treatment response, and patient outcomes [141]. ML algorithms can adapt and improve these models as new data becomes available. As previously discussed, data integration and analytics help discover biomarkers that can guide the development of targeted therapies [142,143]. Precision medicine acknowledges the heterogeneity in patient populations [3], and data-driven methods can account for this variability by estimating inter-individual differences in drug disposition, enabling tailored dosing regimens for diverse patient groups and helping to identify relevant biomarkers or patient characteristics significantly impacting drug PK [144,145].

Integrated data empowers CDS systems, aiding healthcare providers in personalized treatment decisions based on individual patient data and the latest research [146]. This approach enhances early disease detection, tailors treatments, and deepens the understanding of factors influencing health. In PK modeling, data-driven approaches utilize observed data, often from clinical trials or real-world settings, to estimate model parameters governing drug behavior [146]. Unlike theory-driven methods, these approaches prioritize empirical relationships and statistical methods, enabling pharmacometricians to capture complex interactions between drug concentrations and patient-specific factors for more accurate parameter estimates [147]. Nevertheless, there is a symbiotic relationship among data-driven, theory-driven, and hypothesis-driven strategies in scientific research. Data-driven methods offer the advantage of uncovering unexpected insights and patterns that might not be apparent through preconceived theories. Theory-driven approaches provide a theoretical framework that guides research design and interpretation. Hypothesis-driven strategies, in turn, enable researchers to test specific predictions and refine theories. An integrated use of these methodologies enhances the rigor and depth of scientific investigations.

Optimizing clinical trial designs by identifying relevant covariates that influence drug PK streamlines trial enrollment, ensuring the inclusion of precisely the right patients [148], and enhances the accuracy and efficiency of clinical investigations, allowing advances in accurate parameter estimation for drug safety and efficacy [149]. Data-driven methods excel in predicting adverse events, guiding dose adjustments, and minimizing side effects. Rigorous validation and refinement using real-world data reinforces their applicability across diverse clinical settings and patient populations. Their adaptability to changing patient data aligns with the dynamic nature of precision medicine applications [150], optimizing treatment plans and clinical trial designs and leading to cost savings in drug development and healthcare services.

Data-driven approaches involve statistical and computational techniques to collect, analyze, and interpret data, prioritizing the identification of patterns, correlations, trends, and relationships [151]. They are well-suited for handling large datasets, making them valuable in ML, AI, and big data analytics. They can adapt to changing data and evolving insights, allowing dynamic decision-making and the extraction of complex patterns and relationships within PK data. Their strength lies in multivariate analysis, effectively considering numerous variables simultaneously to uncover hidden correlations that classical methods might miss. One real-life example of a hidden correlation that data-driven approaches can identify is the relationship between certain genetic markers and drug efficacy. Classical methods might not detect this correlation due to the complex interplay of genetic factors. These examples illustrate how data-driven approaches can reveal insights that are not immediately obvious, leading to a deeper understanding of complex phenomena and informing more effective decision-making.

By considering individual patient characteristics, data-driven approaches enable the tailoring of drug dosages to maximize therapeutic benefits while minimizing side effects [152,153]. In contrast to traditional approaches, which often require extensive trial-and-error adjustments to find optimal dosages [154,155], these methods accelerate this process, reducing costs and risks, and can be used in early drug development stages to predict PK parameters, facilitating decision-making and dose selection [156,157]. These approaches support adaptive dosing strategies that can be modified in real time based on a patient’s response and changing clinical conditions [158,159].

### 5.2. Challenges and Regulatory Considerations in Data-Driven Pharmacokinetic Modeling

While data-driven approaches offer substantial advantages, they face challenges, such as ensuring data quality, addressing bias in datasets, and interpreting complex ML models. Strategies to mitigate these concerns include: (a) Data preprocessing, which involves cleaning, normalizing, and handling missing values to ensure consistency and quality, removing outliers, addressing imbalanced classes, and ensuring data representativeness; (b) Feature selection, identifying the most relevant features for the model using techniques such as principal component analysis or correlation analysis to reduce the impact of noise and improve the model’s performance; (c) Model evaluation, employing appropriate metrics, such as accuracy, precision, recall, and F1-score to assess the performance of the model on a validation set or cross-validation techniques, preventing overfitting and ensuring effectiveness on unseen data; (d) Hyperparameter tuning, optimizing the hyperparameters of the ML models through techniques like grid searching or random searching to find the best combination of hyperparameters for optimal performance; (e) Ensemble methods, combining multiple ML models to make predictions by aggregating their individual predictions, which can help to reduce the impact of any single model’s errors and improve the overall accuracy; (f) Regulatory guidelines, adhering to regulatory guidelines, such as those set by the FDA, to ensure responsible and compliant use of AI and ML in PK modelling.

The current state of regulatory guidelines for incorporating data-driven models into drug development and clinical practice is still evolving. Regulatory agencies like the FDA have released guidance documents on the use of AI and ML in drug and biological product development, providing recommendations on data quality, algorithm development, and validation. The use of ML/AI-based modeling approaches in PBPK modeling can help support dose selection for future clinical trials or guide drug development strategies. Collaborations between researchers, regulatory agencies, and industry partners can help to advance the understanding of AI and ML applications in PK modelling and address the challenges associated with their implementation. Key challenges and opportunities include: (1) Ensuring data quality and diversity for robust ML model training; (2) Addressing model interpretability to understand the underlying mechanisms and reasoning behind the model’s outputs, especially in the context of drug development and clinical practice; (3) Securing regulatory acceptance by transparently communicating the model’s performance, data quality, and potential limitations; (4) Integrating traditional pharmacometric methods into data-driven models, such as popPK and PD models, to improve overall model performance.

## 6. Artificial Intelligence: Integration of Machine Learning in Pharmacometrics

The introduction of AI to healthcare coincided with the emergence of PK modelling and simulation techniques [160,161]. Since then, AI has played a crucial role in the medical field, particularly in the analysis and processing of complex and diverse healthcare data, such as EHRs. Within AI, ML is a subgroup that has contributed to advancing mathematical and statistical algorithms capable of effectively learning from data to generate predictions and insights [162].

Pharmacometrics and ML are distinct yet complementary approaches to enhance drug therapy and disease management. As previously stated, pharmacometrics utilizes mathematical models to describe the behavior of drugs in the body, the effects of drugs over time, and the variability among individuals and to optimize and predict dosing and outcomes. ML, in turn, is a subset of artificial intelligence that focuses on building systems that learn from data, identify patterns, and make decisions with minimal human intervention. In healthcare, ML algorithms analyzes large datasets to predict disease progression, identify potential drug targets, and personalize treatment plans based on patient characteristics. Pharmacometrics quantitatively describe and predict drug and disease behavior, enabling the optimization of therapeutic strategies. In contrast, ML prioritizes the accuracy of outcome predictions [163]. As Poweleit et al. note, popPK modelling differs from ML in the kinds of models it uses, despite being considered a subset of ML. To ensure the physiological and pharmacological relevance of parameter estimations, popPK modeling depends on structurally grounded models founded in pharmacokinetic and pharmacodynamic concepts [164]. On the other hand, ML is focused on minimizing prediction errors by selecting the most appropriate model from a range of possibilities.

The convergence of pharmacometrics and AI approaches, rooted in the 1990s, when neural networks were first applied to PK/PD analyses [165,166], is gaining momentum. However, the success of both pharmacometrics and ML fundamentally depends on the quality of analysis datasets, which, in turn, hinges on the calibre of reference data sources and the meticulousness of data preparation processes [162]. Pharmacometrics datasets, frequently developed for specific analysis, face challenges in review and exchange due to their unique construction and lack of standardization [167]. By serving as a computational link and enabling the integration of massive data sources into pharmacometrics analysis, ML can overcome these problems.

Integrating ML into pharmacometrics enhances precision dosing for precision medicine, emphasizing synergy rather than the replacement of conventional methods. ML-driven PBPK/popPK models, considering genetics and patient-specific data, enable precise dosing regimens tailored to individual physiology. Thus, this approach improves drug exposure, efficacy, and disease control, improving therapeutic success [168]. ML’s impact on drug development includes predicting metabolism, identifying safety concerns, and reducing side effects [169]. Tailored dosing considering patient variability ensures individualized treatment recommendations, crucial in diverse populations [170]. Personalized models improve the allocation of healthcare resources [132], reduce overmedication risks, and cut costs. These models can also aid in the early stages of drug development by identifying patient subgroups that may benefit most from a new drug. Again, this can streamline clinical trials, improve patient recruitment, and increase the chances of successful drug development [171]. Ultimately, personalized dosing and treatment regimens are aligned with the goal of patient-centered care, fostering satisfaction and adherence to therapies [172].

Traditional PBPK and popPK models often rely on static datasets that may not reflect the latest patient information or trends in health data. ML-driven dynamic data integration enables the continuous flow of real-time patient data into these models. This ensures that the models remain up-to-date and relevant, reflecting changes in patient health status, treatment responses, and demographics as they occur. This timeliness is crucial for optimizing treatment decisions [147,173]. This influx of data can lead to more accurate and comprehensive models and identify subtle patterns and associations that may not be apparent in smaller or less diverse datasets. As a result, ML-driven PBPK and popPK models can be continuously validated against real-world patient data [174]. This validation process ensures that the models are not only accurate in controlled clinical settings but also in the messy and complex environment of real-world healthcare, which enhances confidence in model predictions and their utility in clinical practice [169].

Additionally, ML-based models support adaptive CDS systems, providing real-time recommendations for drug dosing, treatment adjustments, and monitoring based on the latest patient data. The real-time integration of data sources also benefits research and drug development. Researchers can access a wealth of real-world data to study drug responses, patient outcomes, and the impact of treatments on diverse populations. ML-driven data integration can also reduce the administrative burden on healthcare providers due to the automated data processing and model updates [175].

Additionally, ML-driven PBPK models offer a cost-effective alternative to traditional drug development, which involves a substantial amount of experimentation and data collection to understand a drug’s PK [176,177]. They significantly reduce the need for expensive and time-consuming experiments, especially in the early stages. This cost-saving aspect is highly advantageous for pharmaceutical companies and researchers. ML-driven models can uncover complex relationships between drug PK and patient characteristics, such as genetics, demographics, and comorbidities [178]. Moreover, traditional methods for selecting relevant covariates in popPK models often involve manual and time-consuming processes, with pharmacometricians performing stepwise covariate modelling, which entails sequentially testing and adding covariates, leading to an iterative and time-intensive procedure [177]. ML frameworks, on the other hand, can automate and expedite this procedure by simultaneously analyzing a broad range of potential covariates. This not only reduces the time required for model development and refinement, but also handles large datasets and a multitude of potential covariates more comprehensively than manual methods [168]. In fact, this comprehensive exploration increases the likelihood of identifying important covariates that may have been overlooked in a manual approach, since manual stepwise covariate modelling can introduce bias due to the subjective decisions made by modelers during the process [178]. ML frameworks, in contrast, rely on data-driven algorithms that are less prone to these types of biases, resulting in more objective and data-supported covariate selection. Moreover, they can capture nonlinear associations and synergistic effects that may be challenging to detect manually. This enhanced predictive accuracy can lead to better individualized dosing recommendations and improved clinical outcomes. For example, Zhu et al. [179] discuss how ML serves as a rapid screening tool for covariates in popPK models.

ML-driven PBPK models offer a versatile solution to address the variability in clinical practice, demonstrating robust performance across diverse patient populations and medical conditions. As these models learn effectively from extensive patient data, they provide reliable predictions for various groups, from pediatrics to geriatrics, and across different demographics. In addition, the ability to adapt predictions according to new PK data [169] due to changes in physiology and organ function resulting from different diseases and medical conditions is crucial for optimizing drug therapy in patients with various medical conditions, including chronic diseases or infections.

ML-driven PBPK models can account for demographic factors, which are essential for individualizing drug dosages based on patient-specific characteristics, highlighting robust performance across various patient subgroups [180]. This consistent accuracy in predictions for different populations and clinical scenarios, ensuring generalization across diverse contexts, enhances trust and acceptance among healthcare providers and regulators. Therefore, the fact that decision-making on treatment strategies may be supported by this type of model also enables questions related to complex medical cases or rare diseases to be addressed [85], where available clinical data is limited [181].

ML-PBPK/popPK models can also predict drug toxicity and safety profiles more accurately, aiding in the early identification of potential adverse effects during drug development [182,183]. Pharmaceutical companies can decide whether to continue development, modify the drug’s formulation, or explore alternative compounds. Once more, this early detection can save substantial time and resources that might have been invested in less promising candidates, and avoiding late-stage safety issues can significantly reduce the costs of drug development [184]. Identifying safety concerns early can prevent costly clinical trial failures or regulatory setbacks, leading to extensive delays and financial losses [185]. Regulatory agencies, such as the FDA, require thorough safety assessments during drug approval processes [186,187], and ML-PBPK/popPK models can provide valuable insights and data to support these assessments, helping pharmaceutical companies meet regulatory requirements more effectively. The following table (Table 2) summarizes the integration points of ML and phamacometrics.

### 6.1. Examples of ML Approaches That Can Address Unique Challenges and Opportunities within Pharmacometrics

ML empowers PBPK and popPK models with notable benefits. Pioneering work by Woillard et al. utilized extreme gradient boosting (XGBoost) models to predict the exposure of drugs like tacrolimus and mycophenolic acid [188,189,190]. ML algorithms, including classification and regression trees, excel in optimizing doses, especially for drugs with a narrow therapeutic index like vancomycin [191,192]. Applications extend to predicting optimal doses for medications like lamotrigine and warfarin, demonstrating moderate to good accuracy and target attainment rates [193,194]. ML’s pivotal role in enhancing Bayesian approaches within model-informed precision dosing (MIPD) systems is noteworthy.

Gill et al. used regression-based ML to predict drug exposure changes due to interactions [195]. The model, with 78% accuracy within twofold observed changes, highlighted early drug-discovery features for risk assessment. Despite potential biases, it showcased ML’s power in capturing relationships, aiding decision-making in drug discovery [195]. Song et al. focused on DDI prediction using similarity-based ML, achieving an AUROC exceeding 0.97 [196]. Additionally, Minerali et al. compared ML algorithms, with the best Bayesian model, achieving a ROC of 0.814, illustrating ML’s effectiveness in predicting DILI and identifying potential issues in clinical compounds and FDA-approved drugs. These studies collectively demonstrate ML’s versatility and efficacy in pharmacology, advancing early risk assessment and safety evaluation in drug development.

A study developed an ML model predicting methicillin-resistant *Staphyloccocus aureus* (MRSA) infection likelihood in community-acquired pneumonia (CAP) patients within 72 h [197]. Using classification tree analysis, the model achieved high accuracy (ROC area: 0.775), aiding risk stratification for targeted interventions. Despite promising results, limitations include a small sample, lack of external validation, and interpretability concerns due to the “black box” nature of ML models. Further validation is crucial for real-world reliability and practicality.

Finally, Harun et al.’s study focuses on methodological considerations in ML-based exposure–response analysis [190]. The study underscores the importance of following proposed ML workflow practices, including SHAP analysis, hyperparameter tuning, and model reliability checks. Failure to adhere to these practices can lead to errors and confidence interval issues. The study showcases XGBoost’s potential in accurately estimating exposure–response relationships. Exposure–response analysis in pharmacometrics is vital for drug development, optimizing therapeutic outcomes, and ensuring patient safety. Liu et al.’s study evaluates ML-based techniques in this type of analysis, highlighting their potential in handling complex datasets and identifying confounding factors [196,198]. The combination of ML and PK approaches has demonstrated reduced mean percentage errors and prediction errors compared to using only the maximum a posteriori method. ML frameworks have also facilitated efficient covariate modelling in popPK models, enabling faster and more streamlined selection of relevant covariates while maintaining computational efficiency. In supporting EHR systems and data collection, ML has proven useful in automating data extraction, processing, and preparation for PK analyses. ML-based systems have been developed to extract structured and unstructured EHR data, reducing the time and effort required for popPK analysis. These systems have efficiently formatted data for analysis using PK software like NONMEM. With the increasing availability of big data, there is a growing interest in leveraging ML (and AI) to enhance patient outcomes in drug therapy. Indeed, ML and AI are essential bridges between big data and pharmacometrics, facilitating efficient analysis and interpretation of vast information.

PBPK models are on the verge of expanding their capabilities to manage population-level data and create population-specific PBPK models routinely. Similarly, there are expectations for some level of automation in system pharmacology models. ML is expected to be central in bringing these subfields together, fostering smooth collaboration and integration. This paradigm shift in pharmacometrics reverberates across the broader pharmaceutical industry. Professionals in this sector are poised to transition into the role of model interpreters as ML algorithms progressively shoulder the computational workload [199]. Looking forward, the role of ML in this field is set for significant evolution. Prominent voices in the field have alluded to the transformation awaiting the landscape of pharmacometrics [200,201]. ML techniques are set to automate various facets of the process, such as popPK models, driven by methods like genetic algorithms [202,203]. However, it is imperative that a fundamental understanding of these algorithms and their limitations is retained. Interestingly, Kolluri et al. emphasize the importance of recognizing that the scientific method is not obsolete when making inferences about data and that informed decision-making on the optimal use of AI/ML in drug development is necessary [157]. A landscape analysis of regulatory submissions to the FDA reveals a rapid increase in AI and ML applications since 2016, with a particularly significant rise in 2021. This trend emphasizes the need for standards and best practices to guide and ensure the proper implementation of AI and ML applications in healthcare [204]. The International Coalition of Medicines Regulatory Authorities (ICMRA) has also published recommendations for stakeholders regarding the uses and challenges of AI in drug development, which the European Medicines Agency has endorsed [204]. As new applications and approaches emerge, the guidelines for AI and ML in healthcare will continue to evolve to address the specific needs and challenges of the field. However, limitations and challenges remain (Table 3).

### 6.2. Challenges and Future Directions

Navigating the intricate landscape of integrating ML with PBPK (ML-PBPK) and popPK (ML-PopPK) models presents many complex challenges and opportunities in pharmaceutical research and precision medicine. Concerning data integration and quality, pharmaceutical research relies on data from various sources, including clinical trials, EHRs, wearable devices, and omics data [205]. Each source may have different formats, standards, and levels of quality. Combining them into a cohesive dataset for ML modeling can be challenging. Thus, standardized data integration pipelines are required, addressing formats and maintaining compatibility. Implementing standard data formats, such as the CDISC (Clinical Data Interchange Standards Consortium), can facilitate this standardization process [206].

The accuracy and reliability of ML-driven models heavily depend on high-quality input data. To ensure this, preprocessing techniques are usually employed, particularly outlier detection, missing data imputations, and cleaning [207], as well as regular audits and validation checks to identify and rectify data quality issues without neglecting metadata management [208]. Establishing a robust metadata management system helps in tracking the lineage of data and assessing its reliability for modeling purposes.

Integrating data from various sources may involve sensitive patient information. Maintaining data privacy and complying with regulations like the General Data Protection Regulation (GDPR) and Health Insurance Portability and Accountability Act (HIPA) is essential. Anonymization and de-identification techniques can be employed to protect patient privacy while integrating data [205,209].

In addition to standardization, harmonizing data is a crucial step, particularly when dealing with patient-specific information. This involves reconciling differences in terminologies, units of measurement, and data collection methods to ensure the comparability and effective utilization of data. To ensure that different systems and platforms can interact and share data seamlessly, the development of application programming interfaces (APIs) and data exchange standards can facilitate interoperability [210]. Establishing clear data governance policies and practices is essential for managing data integration and quality. This includes defining roles and responsibilities, data stewardship, and data lifecycle management to maintain its integrity over time. ML models are dynamic and evolve with new data. Implementing data versioning protocols ensures that changes in data sources and quality are tracked and model updates can be managed effectively. For this reason, collaboration between data scientists, domain experts, clinicians, and information technology (IT) professionals is necessary to effectively address data integration challenges [211].

Moreover, patient data must be handled with utmost privacy and security. However, diversity, equity, and inclusion (DEI) and concern for bias are also critical considerations in the integration and analysis of diverse datasets in precision medicine. Biases can derail any attempt to improve the culture of DEI, and this is particularly relevant in healthcare. Human beings have inherent biases, and these biases can manifest in the collection, analysis, and interpretation of data, potentially culminating in disparities in healthcare access, treatment, and outcomes, particularly for underrepresented minority populations. Therefore, it is essential to consider DEI and bias in the integration and analysis of diverse datasets in precision medicine and to implement strategies to mitigate them, such as using diverse study populations, validating biomarkers across diverse populations, and using rigorous statistical methods to analyze data. Diversifying the composition of healthcare providers and research teams is one strategy to address DEI in precision medicine. Studies have demonstrated that underrepresented minority physicians and women are more likely to provide care to underserved populations and to address health disparities. Additionally, community engagement and education programs can help increase diversity in clinical trials and improve representation of underrepresented groups in research [212]. ML models can inadvertently perpetuate biases present in the training data. Mitigating bias and ensuring fairness in predictions, especially in healthcare decisions, is an ethical imperative [213]. For instance, Lee et al. demonstrated the significance of integrating ML techniques with robust de-identification methods to safeguard sensitive healthcare information. Advanced models can play a pivotal role in verifying the accurate application of de-identification techniques, thereby promoting both data confidentiality and usability while aligning with ethical and regulatory standards [214].

The opacity of ML models, that is, the fact that they are often considered “black boxes”, can hinder their adoption in clinical practice [215]. Physicians and healthcare professionals need to understand the rationale behind a model’s predictions to make informed decisions regarding patient care, and for this reason, a lack of interpretability can result in mistrust and reluctance to use ML-driven recommendations [216]. Therefore, transparent explanations for model predictions must be provided to solidify trust and empower patients to participate in their own care decisions [217,218]. Implementing model interpretability techniques like SHAP analysis can provide insights into model decision-making [219,220].

Patient-specific predictions should be actionable in a clinical setting, and this requires developing robust models [221,222] that align with clinical workflows and provide practical guidance to healthcare providers [223]. However, this exercise is quite complex due to substantial variability in response to drugs. Failing to account for such heterogeneity may lead to suboptimal treatment outcomes for specific patient groups, affecting its generalizability. Thus, ensuring that the model can handle healthy and diseased populations is essential for its clinical relevance [224,225]. One solution could be to continuously validate models across various patient subgroups and update them as needed.

Validation should encompass diverse datasets, including independent datasets not used during model training [226], to verify the reliability and robustness of ML-enhanced PBPK/popPK models. Models should be evaluated for their long-term predictive performance. This is particularly relevant in chronic diseases where treatment effects may evolve over time because it is crucial to ensure that the model remains accurate over extended periods. Therefore, rigorous validation approaches, including external validation using independent datasets can be helpful.

In addition, ML algorithms can be computationally demanding, particularly when dealing with large datasets or complex models. Efficient algorithm design, parallel computing, and graphics processing unit (GPU) acceleration can optimize resource usage [227]. ML models should be designed to scale with growing data volumes and computational demands, and therefore cloud computing resources are usually employed, which offer scalability and cost-effectiveness, especially for resource-intensive tasks like deep learning [228].

ML algorithms often require large sample sizes, which may exceed what is typically needed for the clinical application of AI and ML. Collaborative efforts are essential to develop comprehensive databases and enhance data quality. AI and ML should complement, not replace, traditional pharmacometrics. Ongoing advancements and collaborations are expected to drive the evolution of precision medicine, with a focus on research, validation, and integration into clinical practice. The future of ML-PBPK/popPK integration holds promise in various strategic directions:

- Expanded applications of PBPK models, informing clinical study design and predicting drug interactions.
- Pediatric dosing regimen prediction to ensure safer and more effective treatments for pediatric patients.
- Utilization of PBPK models for predicting drug exposure in patients with organ impairment.
- Estimation of maternal–fetal drug disposition during pregnancy.
- Prediction of pH-mediated drug interactions using PBPK models.
- Improved predictive performance of popPK models by focusing on data adequacy.
- Integration of generic PBPK models for extrapolations and continuous updates.

## 7. Digital Health and Wearable Technologies

As technology advances, digital health and wearable technologies have increasingly become integrated into patient care. Digital health has gained significant momentum due to several key factors and, besides improving access to healthcare, this discipline also mitigates any inefficiencies in the healthcare system, improves the quality of care, reduces the costs associated with healthcare, and offers more individualized care tailored to patients’ needs [229].

Digital health, a term that refers to the application of information and communication technologies in the medical field and other health professions, plays a major role in precision medicine. It provides the essential tools and technologies for the collection, analysis, and effective application of individualized patient data. This field is constantly developing and has a broad scope, making use of digital technologies such as wearable devices, mobile health, telehealth, health information technology, telemedicine, apps, sensors, data analysis, and other digital solutions to improve the delivery of healthcare services, raise the quality of patient care, and optimize healthcare management [230,231]. For example, the use of digital devices such as smartphones not only facilitates communication but also provides a wide range of applications capable of monitoring blood pressure, recording blood glucose levels, ensuring adherence to drug treatment, and tracking levels of physical activity [231]. These capabilities demonstrate that the adoption of digital medicine enables patients to monitor their health and well-being more precisely, collecting real-time data and making it an essential pillar in contemporary medical practice [232,233]. Another goal of digital health is improving the experience of each patient, as well as the experience of the doctor and other non-medical providers. The final objective is to address health disparities and improve them with an individualized view of each patient [234,235].

Remote sensing and wearables, telemedicine and health information, data analytics and intelligence, predictive modeling, health and wellness behavior modification tools, bioinformatics tools (-omics), medical social media, digitized health record platforms, physician–patient portals, do-it-yourself (DIY) diagnostics, compliance and treatments, decision support systems, and imaging are included in the categories of products and services that digital health has to offer [229].

The continuous collection of individual biomedical data, such as genomics, proteomics, mobile health data, and EHRs, is fundamental. This data is essential for understanding a patient’s unique characteristics and genetic predisposition to disease. This database may be then subjected to advanced analysis using techniques such as ML and AI to identify patterns, trends, and associations in large volumes of patient data, which can be used to personalize treatments [236,237]. A notable example of this process is genomic medicine. Digital health makes it possible to sequence a patient’s genome more affordably and efficiently. This means that doctors can analyze a patient’s DNA to identify genetic variations that can influence the response to specific drugs and treatments [236]. Based on the data collected and the genetic information, doctors can then tailor treatments according to each patient’s specific needs, including the choice of drugs, dosages, and treatment strategies. In addition, digital health may be used to predict individual disease risks based on these data, allowing doctors to develop targeted prevention strategies. To keep a close eye on a patient’s progress, such devices, including wearables and medical sensors, enable continuous health monitoring. This not only helps with current treatment but also with the early identification of health problems. Patients are involved in their own care through health apps and online platforms, allowing them to monitor their progress and make informed decisions [236].

Moreover, digital communication technologies facilitate the exchange of information between clinicians and patients, enabling more effective communication and the sharing of relevant data. This reduces the occurrence of unwanted side effects and leads to better prevention and a more comprehensive approach to patient well-being [234].

The EHR is a digital system that stores medical information and patient health information (e.g., medical history, test results, prescriptions, allergy information) in electronic format (Table 4).

Taken together, the incorporation of EHRs and omics data into digital health enables a more personalized, evidence-based approach to healthcare, improving the diagnosis, treatment, and prevention of disease. This convergence represents a significant advance in the ability to use digital information to improve people’s health and well-being.

Further, wearable devices play a fundamental role in digital health, offering a variety of functions that help monitor the health and well-being of individuals (Table 5).

These examples illustrate the diversity of wearable devices in the area of digital health. Each of these devices is designed to meet specific health monitoring and care needs, and many of them are constantly evolving as technology advances. These devices play an important role in collecting real-time data, supporting medical diagnoses, promoting a healthy lifestyle, and improving healthcare [171]. In the context of wearable devices, the variability in sensors and inconsistency in data collection pose challenges in coordinating and assessing quality. User-related issues significantly impact the reliability of digital health data. These include digital health service accessibility, accuracy of the data, consistency of data input by users, and contextual validity of data to relevant aspects. Addressing these issues is crucial for improving the quality, usability, and acceptability of digital health interventions [56].

Furthermore, precision medicine, driven by the collection and analysis of this data, promotes innovation and collaboration in digital health technologies. The idea of multidisciplinarity continues to be essential. Facilitating the integration of personalized biomedical data collection and precision medicine into society requires public education, training for healthcare professionals, collaboration between experts in different areas, clear regulations, financial incentives, academic partnerships to drive innovation and develop patient-friendly technologies, and universal access and respect of ethical principles.

It is also important to ensure data security, privacy, and interoperability for effective integration into precision medicine workflows. This involves implementing robust security measures, appropriate regulation, and informed consent from patients for the use of their data. By doing so, we can harness the potential of digital health technologies and wearable devices to improve the quality of healthcare and promote a more personalized, evidence-based approach to medical treatment [238].

## 8. Clinical Trials and Study Design

The design of clinical trials has experienced a profound transformation in response to the medical paradigm shift, which recognizes that traditional one-size-fits-all treatments are often ineffective or produce negative effects in patients [239,240]. Hence, the next generation of trials must be a symbiosis between patient-centered strategies, where the therapeutic interventions are tailored to patient-specific biomarkers, and conventional drug-centered strategies, focused on evaluating the efficacy, safety, and pharmacological properties of the drug under study.

In fact, driven by fast advancements in omics, recent biomarker-based clinical trials have emerged as a very promising approach in this new era. In addition, advanced computational tools have revolutionized the way clinical trial data is analyzed. There is a wide variety of in silico methods that allow accurate data processing.

**Figure 4:**
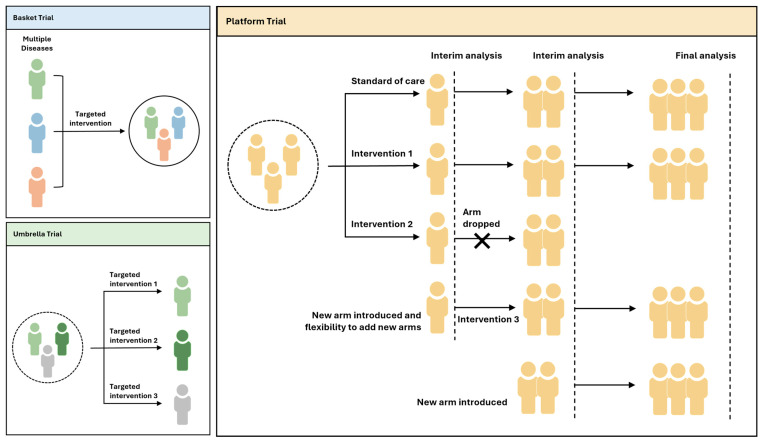
Representation of basket trials, umbrella trials, and platform trials. Created with SMART—Servier Medical ART. Available online: https://smart.servier.com (accessed on 22 September 2023).

As patient-centered trial designs, master protocols have arisen, classified into basket trials, umbrella trials, and platform trials (Figure 4) [241,242,243,244]. They have been increasingly implemented, particularly in the field of oncology. According to Park et al. [241], at the time of publication (2019), there has been a rapid increase in the number of master protocols. Basket trials consist of evaluating a targeted therapy against multiple diseases sharing common molecular alterations. Umbrella trials, on the other hand, involve multiple interventions for a single disease stratified into subgroups according to molecular alteration. In turn, platform trials evaluate several treatments against a common control group [240,245,246,247]. Another innovative approach is adaptative design, which enables the dynamic evolution of studies [159]. This method allows for the early discontinuation of ineffective treatment arms while increasing randomization to more promising therapies. Nevertheless, some limitations associated with the early elimination of treatment may be listed, including the lack of consistent data on safety.

Although less common, home-based clinical trials are being conducted [248], especially in patients with cancer and limiting diseases. This site-less clinical trial design simplifies patient recruitment, enables the inclusion of more diverse populations, and increases participants’ enrolment rates. However, these trials also pose some challenges and risks in terms of data reliability. The quality of data collected from home-based clinical trials depends on the validity and reliability of the instruments used for data collection, such as questionnaires, diaries, sensors, or devices. These instruments need to be designed carefully to ensure that they capture the relevant aspects of the patient’s situation and outcomes and that they are easy to understand and complete by the patients. They also need to be tested for accuracy, completeness, consistency, and contextual validity before being used in clinical trials. The security of data collected from home-based clinical trials is crucial to protect the privacy and confidentiality of the patients. Researchers need to work with information technology professionals to ensure that data collection is safe and secure while maintaining patient privacy during decentralized trials. This may involve using encryption, authentication, authorization, backup, and recovery methods [249].

Another emerging trend in the clinical trial landscape involves the integration of digital health technologies, such as mobile devices, mobile apps, and remote monitoring devices, directly into the study’s framework. These innovative studies are often referred to as virtual clinical trials (VCTs), as they leverage digital tools to remotely gather data from participants instead of requiring in-person visits to research facilities. There are several digital tools currently available: eConsent, a digital method of obtaining informed consent from participants; electronic patient-reported outcomes (ePRO), which represent health-related outcomes (symptoms, adverse effects) directly reported by the patient and collected electronically; and sensors and wearable devices [250]. The most evident advantage of these studies is the increased participant adherence, as they participate from the convenience of their home. Moreover, data collected through digital devices may enable continuous real-time data acquisition rather than periodic data collection during in-person visits.

The ease of digital data collection has led to a huge amount of information, requiring complex and time-intensive analysis [240]. The analysis of real-world data using advanced computer methods provides real-world evidence. This is where AI and ML algorithms can be used to address this challenge and rapidly discover new therapies. Interestingly, real-world data has already practical uses, since the FDA has approved at least two cancer drugs developed using it [251,252]. Also, real-world data holds particular significance in assessing drug efficacy and safety in patient populations frequently excluded from randomized clinical trials, such as patients with limited performance status, older patients, patients with serious comorbidities, or underserved populations who may not be able to travel to an academic centre for a clinical trial [240].

In summary, these clinical trials offer great potential for improving treatment outcomes and reducing adverse effects, ultimately leading to a transformative era of personalized healthcare. There are still, however, opportunities for further evolution.

## 9. Future Perspectives: Integration of In Silico Tools in Hospital Settings

Expectation arising from the application of computational methods, such as ML, deep neural networks, and multi-modal biomedical AI, has been the reinvigoration of clinical research, including drug discovery, image interpretation, streamlining EHRs, improving workflow, and, over time, advancing public health [239]. Therapeutic monitoring and clinical decision-making constitute a multifaceted process in today’s hospital settings. Healthcare professionals employ clinical assessments, interacting with patients to collect vital information about their medical history and current symptoms, guiding subsequent steps. We believe that we are moving towards a reality in which all these technologies will be applied in the context of clinical practice. However, before that, there are issues that must be overcome.

Collaborative care teams, comprising various healthcare professionals, including doctors, nurses, pharmacists, and specialists, collaborate on patient care plans to leverage collective expertise for more informed clinical decisions. Since patient engagement is a fundamental aspect of modern healthcare, hospitals need to actively involve patients in their care by providing information, discussing treatment options, and considering individual preferences during decision-making.

Despite exponential growth in acquiring healthcare data, the capacity to integrate such data to improve health outcomes presently fails to meet technological advances. These challenges can be overcome with the use of AI and other computing technologies in health services workflows. Genomic sequencing, providing detailed information about an individual’s genetic makeup, has particularly benefited oncology and genetics, especially because of the several approvals for biomarker-based targeted therapies and immunotherapy [229,253,254]. Omics-based assays can be used to study the complex interactions in severe diseases, facilitating early-stage intervention and the selection of the most fit treatment.

EHRs streamline the management of health data by providing a centralized, comprehensive, and up-to-date repository of patient information [255,256]. This potentially eliminates the need for paper-based records, reducing errors associated with manual data entry and retrieval. Therefore, EHRs contribute to the creation of medical knowledge in two ways: (a) they enable the aggregation and analysis of large volumes of patient data for research, epidemiological studies, and the discovery of patterns and trends that inform medical practices; (b) CDS provides evidence-based guidelines and alerts to clinicians, contributing to better health outcomes. As such, EHRs are fundamental to the advancement of precision medicine. Key advantages in this context include genomic integration with clinical data and facilitation of personalized treatment plans by providing a comprehensive view of a patient’s medical history, lab results, and other data. This enables healthcare providers to select treatments that are most likely to be effective for a specific patient.

Interoperability is critical for EHRs to fulfill their potential and ensure continuity of care. Patients can receive consistent care even if they change healthcare providers or facilities once their records can be accessed and updated from different systems. Unfortunately, non-interoperability is currently one of the biggest limitations on the exchange of data between different systems.

Alongside the uniformization of EHRs, the integration of CDS systems in hospitals’ workflows is crucial in precision medicine. The healthcare providers need real-time, data-driven recommendations based on patients’ specificities, including genomic data, risk assessment and predictive modelling, DDI alerts, clinical guideline adherence, continuous learning and improvement, and patient engagement. However, besides an infrastructural challenge, the use of CDS systems also demands training staff to accurately introduce and interpret information; keeping CDS systems up-to-date with evolving medical knowledge and technology can be resource-intensive, and evaluating the actual impact of CDS systems on patient outcomes can be challenging.

Therapeutic drug monitoring (TDM) is particularly important for medication management, involving regular assessments of drug levels in the bloodstream to optimize dosages and ensure safe and effective treatment. For example, in the case of a patient taking anticoagulant medication, regular TDM may be conducted to ensure the medication is within the therapeutic range and effectively prevent blood clots. In this context, pharmacometrics is a powerful tool. Besides this, the integration of pharmacogenomic data with pharmacometric models allows for even more precise dosing recommendations based on a patient’s genetic profile.

All stakeholders must be involved in change. Healthcare providers need to develop trust in the information generated by AI applications (and other computational methods) and to face AI and advanced robotic systems as professional partners; legislators must speed up regulatory policies that clarify boundaries and guarantee patient safety and privacy; IT departments are critical to ensure robust IT infrastructure to support data transfer, integration, and analysis as well as to integrate CDS systems into EHR systems for seamless use in clinical workflows. Nowadays, clinical workflows do not consider big data-driven approaches. Consequently, the main priorities for the integration of in silico tools in hospital settings are the development of IT infrastructure capable of aligning big data with clinical practice, standardized protocols and boundary-setting, and the design of comprehensive training programs for healthcare professionals.

## 10. Conclusions

The integration of omics, biomarkers, pharmacometrics, ML, and digital wearables into healthcare presents a transformative potential for precision medicine and patient care. These methods offer numerous advantages, such as improved diagnostic accuracy, tailored treatment plans, and enhanced patient monitoring.

Among these methods, in silico approaches, which involve computer simulations and modeling, are gaining traction, reducing the need for costly and time-consuming physical examination, speeding up drug development, and enhancing disease understanding. They also hold promise for conducting virtual clinical trials, which can streamline the evaluation of medical interventions. User-friendly, seamless integration with existing healthcare systems and clear insights are crucial for broad adoption.

The education of clinicians and patients in the interpretation of genomic data and the use of wearable technologies is crucial for the successful implementation of these methods. Specialized training modules, integration with EHRs, and informative informatic systems can play a key role. These systems, accessible anytime and anywhere, can include interactive elements, making them cost-effective alternatives to traditional courses. Collaboration with healthcare professionals, educators, and technology experts will be essential to developing systems that meet the needs of both clinicians and patients.

Ethical considerations, funding, and legislative changes are indeed significant factors that could influence the adoption of these healthcare technologies. Ethical challenges include ensuring patient privacy, data protection, and equitable access to these technologies. Adequate funding is necessary to support the development and implementation of these methods, while legislative changes may be required to address regulatory and compliance issues. It is essential for stakeholders to work collaboratively to navigate these challenges and create an environment that supports innovation while safeguarding ethical principles and patient rights.

## Figures and Tables

**Table 1:** Some new therapeutic molecular personalized medicines approved by the FDA [34,35].

Products	Therapeutic Indication
	Cancer
	Abecma (multiple myeloma)
	Exkivity (lung cancer)
	Lumakras (lung cancer)
	Jemperli (endometrial cancer)
	Rybrevant (lung cancer)
	Scemblix (myeloid leukaemia)
	Tepmetko (lung cancer)
	Truseltiq (cholangiocarcinoma)
	Rare Diseases
	Amondys (muscular dystrophy)
	Evkeeza (homozygous familial hypercholesterolaemia)
	Nexviazyme (Pompe disease)
	Nulibry (molybdenum cofactor deficiency)
	Vyvgart (Myasthenia Gravis)
	Welireg (von Hippel–Lindau)
	Other Diseases
	Bylvay (progressive familial intrahepatic cholestasis)
	Cabenuva (HIV-1)
	Leqvio (hypercholesterolaemia)

**Table 2:** Integration points between ML and pharmacometrics.

Integration Points	Details
Data analysis	ML processes big data efficiently, improving patient outcomes in drug therapy. It identifies salient variables and delineates their interdependencies.
Predictive capabilities	ML algorithms excel in predictive capabilities, aiding pharmacometrics in understanding dose–exposure relationships (pharmacokinetics) and exposure marker effects (pharmacodynamics).
Complementing pharmacometric modelling	ML acts as a computational bridge, leveraging its flexibility to complement the complexity of principled pharmacometric modelling, resulting in synergistic effects in pharmacological applications.
Robustness of datasets	ML implementation in pharmacometrics requires robust datasets for training and testing, capturing the distribution of intrinsic and extrinsic factors of interest.
Overfitting	Evaluation data should not be used for training to prevent overfitting, ensuring the model generalizes well to unseen observations and doesn’t fit the training data perfectly.

**Table 3:** ML approaches that can address unique challenges and opportunities within pharmacometrics [157].

Opportunities	How to Address Them?
PKPD model personalization	Developing ML techniques for efficient personalization of PK/PD models to individual patients using sparse data. Integrating patient-specific data, such as genetics, biomarkers, and historical treatment responses to improve model predictions and treatment optimization Predicting the probability of a drug’s success and identifying patient subgroups for maximum therapeutic benefit. **Challenges**: Determining appropriate endpoints and predicting success in pivotal trials. Unsupervised learning can be used for patient clustering to optimize clinical development.
Data integration for rare events	Designing models that integrate information from various sources (EHRs, social media, and wearable devices) to predict and manage rare adverse events not well-captured by traditional pharmacometrics models. **Challenges**: Scarcity of labeled data since rare events occur infrequently.
Adaptive clinical trials that can dynamically adjust treatment regimens based on real-time data analysis	Using ML as an assisted tool for clinical trial oversight, providing efficient ways to protect patient safety, reduce trial duration, and lower costs in clinical trial oversight. **Challenges**: Ensuring data quality and integrity when incorporating data from multiple sources.
Real-world evidence analysis	Using real-world evidence data to refine pharmacometrics models, accounting for patient heterogeneity, treatment variability, and long-term outcomes not adequately captured in controlled clinical trials. **Challenges**: Ensuring data quality and consistency.
Interpretable AI for decision Support	Developing interpretable ML models for transparent clinical decision-making. **Challenges**: Balancing model complexity and transparency; difficult interpretation potentially hindering their acceptance in clinical settings.
Uncertainty quantification	Enhancing pharmacometric models by incorporating uncertainty estimation techniques from ML, providing clinicians with confidence intervals for predictions and allowing for better risk assessment.
Multi-modal data fusion	Investigating methods to effectively fuse data from diverse modalities, such as genomics, proteomics, and imaging data, to create comprehensive patient profiles that can better inform treatment decisions. Breakdown of the multi-modal data fusion process: (1) data collection; (2) data preprocessing; (3) feature extraction and selection; (4) data fusion; (5) model development; (6) model validation; (7) clinical application; and (8) continuous learning (as new data becomes available, the models can be updated and refined, embodying the principles of continuous learning and improvement).
Longitudinal data analysis	Developing models for analyzing longitudinal data over extended periods to capture changes in patient response to treatments.
Ethical and regulatory Considerations	Addressing ethical implications and regulatory challenges of incorporating ML into pharmacometrics, including issues related to data privacy, bias, and validation.
Optimization of drug combination	Exploring ML algorithms to optimize drug combinations by predicting synergistic effects, potential adverse interactions, and tailoring treatments for individual patients.

**Table 4:** The uses and benefits of EHRs. Adapted from [168].

EHR Benefits	Integration of EHR in Healthcare
Information access and sharing	EHRs facilitate quick and secure access to patients’ medical information, allowing healthcare professionals to make informed decisions and order care.
Better care management	EHRs help you better manage the care of chronic patients by enabling continuous monitoring and adjustment of treatment plans based on real-time data.
Integration and coordination	The integration of RSE (remote sensing and earth observation) into healthcare systems allows for more efficient coordination between different healthcare providers, improving continuity of care.
Clinical research	RSE data can be used in clinical research to identify health trends, evaluate the effectiveness of treatments, and improve evidence-based medicine. Furthermore, omics data, which encompasses genomic, transcriptomic, proteomic, and metabolomic information, plays a crucial role in precision medicine. This data enables the personalization of treatments based on the genetics and individual characteristics of each patient, improving the effectiveness of care. Omics data analysis also helps identify genetic markers of diseases, enabling early prevention and diagnosis.

**Table 5:** Wearable devices used in digital health and their uses. Adapted from [168].

Wearable Devices	Properties, Capabilities, and Applications
Smartwatches	Monitor heart rate, measure blood pressure, track physical activity, count steps, monitor sleep quality, and send reminders to move, drink water or perform exercises
Fitness trackers	Monitor steps, distance traveled, calories burned, heart rate, and even track specific exercises like running and swimming
Glucose-monitoring devices	For people with diabetes, devices such as continuous glucose monitors (CGM) offer the ability to monitor blood glucose levels in real-time. They can send alerts when glucose levels are out of ideal range
Portable electrocardiogram (ECG) devices	Some smartwatches can perform ECGs. They can detect abnormal heart rhythms, such as atrial fibrillation
Sleep-monitoring devices	These devices record sleep patterns, duration, and quality. They provide insights into improving sleep habits
Breath-monitoring devices	Can monitor respiratory rate and blood oxygen saturation. This is useful for monitoring breathing problems such as sleep apnea
Virtual and augmented reality (VR/AR) Devices	In rehabilitation areas and therapy, VR and AR devices create virtual environments for therapeutic purposes, such as rehabilitation after injuries or strokes
Smart glasses	These are used in medical settings for access to clinical information, real-time documentation, and telehealth
Physiological activity-monitoring devices	In addition to the most well-known devices, some wearables monitor specific physiological activities, such as body temperature, exposure to UV light, hydration, and much more
Wearable sensors for clinical research	In clinical research, wearable sensors are used to collect objective and accurate data about the health of patients in clinical studies, enabling a deeper understanding of different medical conditions
Augmented reality glasses for surgery	In medicine, augmented reality glasses are used by surgeons to provide real-time information during surgical procedures, making them more accurate and safer

## Data Availability

Not applicable.
